# Hepatic Progenitor Cells Contribute to the Progression of 2-Acetylaminofluorene/Carbon Tetrachloride-Induced Cirrhosis via the Non-Canonical Wnt Pathway

**DOI:** 10.1371/journal.pone.0130310

**Published:** 2015-06-18

**Authors:** Jiamei Chen, Xiao Zhang, Ying Xu, Xuewei Li, Shuang Ren, Yaning Zhou, Yuyou Duan, Mark Zern, Hua Zhang, Gaofeng Chen, Chenghai Liu, Yongping Mu, Ping Liu

**Affiliations:** 1 Shuguang Hospital affiliated to Shanghai University of Traditional Chinese Medicine, Institute of Liver Diseases, Shanghai University of Traditional Chinese Medicine, Shanghai, China; 2 Department of Internal Medicine, Institute for Regenerative Cures, University of California Davis Medical Center, Sacramento, California, United States of America; Shanghai University of Traditional Chinese Medicine, CHINA

## Abstract

Hepatic progenitor cells (HPCs) appear to play an important role in chronic liver injury. In this study, cirrhosis was induced in F-344 rats (*n* = 32) via subcutaneous injection of 50% carbon tetrachloride (CCl_4_) twice a week for 8 weeks. Then, 30% CCl_4_ was administered in conjunction with intragastric 2-acetylaminofluorine (2-AAF) for 4 weeks to induce activation of HPCs. WB-F344 cells were used to provide direct evidence for differentiation of HPCs to myofibroblasts. The results showed that after administration of 2-AAF, the hydroxyproline content and the expressions of α-SMA, Col I, Col IV, TGF-β1, CD68, TNF-α, CK19 and OV6 were significantly increased. OV6 and α-SMA were largely co-expressed in fibrous septum and the expressions of Wnt5b, frizzled2, frizzled3 and frizzled6 were markedly increased, while β-catenin expression was not statistically different among the different groups. Consistent with the above results, WB-F344 cells, treated with TGF-β1 in vitro, differentiated into myofibroblasts and α-SMA, Col I, Col IV, Wnt5b and frizzled2 expressions were significantly increased, while β-catenin expression was decreased. After blocking the non-canonical Wnt pathway via WIF-1, the Wnt5b level was down regulated, and α-SMA and F-actin expressions were significantly decreased in the WIF-1-treated cells. In conclusion, these results indicate that HPCs appear to differentiate into myofibroblasts and exhibit a profibrotic effect in progressive cirrhosis via activation of the non-canonical Wnt pathway. Blocking the non-canonical Wnt pathway can inhibit the differentiation of HPCs into myofibroblasts, suggesting that blocking this pathway and changing the fate of differentiated HPCs may be a potential treatment for cirrhosis.

## Introduction

Sustained injury causes hepatocyte senescence in chronic liver disease when mature hepatocyte proliferation is exhausted and suppressed [1, 2]. This condition results in activation of hepatic progenitor cells (HPCs), known as oval cells in rodents or hepatic stem/progenitor cells in humans. Identification and characterization of HPCs, and their differentiation into functionally mature liver cells, is an evolving goal in stem cell therapy; however, there are conflicting findings concerning the role of stem cells in liver fibrosis and cirrhosis. In some cases, stem cells transplanted into rats or mice with hepatic fibrosis are differentiated into hepatic stellate cells (HSCs) and myofibroblasts, resulting in promoting the fibrotic progress [3, 4]. In contrast, if the pathological microenvironment is improved through therapy or by removing the injury, stem cells may differentiate into hepatocytes, thus promoting injury repair and regeneration [5, 6], though the mechanism by which this occurs is unclear.

The complex signaling pathways that regulate HPC proliferation have been identified in HPCs themselves and in adjacent cells, which may be present within the HPC microenvironment [7]. Intracellular signaling by Wnt ligands is transduced via two different pathways: the “canonical pathway”, which involves regulation of β-catenin and the “non-canonical pathway”, which is independent of β-catenin [8]. Several rodent studies have shown that the non-canonical Wnt pathway plays an important role in HPC expansion and differentiation into hepatocytes [9, 10]. Non-canonical Wnt pathway activation has been observed in the HPC differentiation into myofibroblasts [11]. However, the relationship between HPC differentiation and Wnt signaling is not clear in the pathological microenvironment of progressive cirrhosis.

In the present study, a rodent model of cirrhosis was developed. Cirrhosis was induced by subcutaneous injection of carbon tetrachloride (CCl_4_). During the period of cirrhosis progression, CCl_4_ was administered subcutaneously in conjunction with 2-acetylaminofluorine (2-AAF) and the effect of progressive cirrhosis on differentiated HPCs was observed. The signaling pathway(s) related to the effect of cirrhosis on HPCs was also examined. WB-F344 cells were also used to investigate the differentiation pathways in order to confirm the *in vivo* findings.

## Methods and Methods

### Animals

Female Fischer 344 (F344) rats (*n* = 32), aged 7–8 weeks and weighing 180–200 g, were obtained from The Shanghai Experimental Animal Center of the Chinese Academy of Sciences (Shanghai, China). Animals were housed in a constant temperature, under a 12-hour dark/light cycle and supplied with laboratory chow and water ad libitum. All animal experiments were approved by Shanghai University of Traditional Chinese Medicine’s Animal Ethics Committee (Guide for Animal Experiments, Shanghai University of Traditional Chinese Medicine) (NO.20130132).

### Induction of cirrhosis and HPC activation/proliferation

Cirrhosis was induced by subcutaneous injection (2 ml/kg body weight) of a 1:1 solution of CCl_4_ and olive oil, administered twice a week for 8 weeks (8wM, *n* = 3). At the beginning of the 9^th^ week, the dose was changed to a 3:7 solution of CCl_4_ and olive oil (2 ml/kg) and was administered for another 4 weeks in order to maintain cirrhosis progression and reduce mortality. The rats were randomly divided into groups as follows: CCl_4_ injection only (12wM, *n* = 8), CCl_4_ plus intragastric administration of a low dose of 2-AAF (5 mg•kg^-1^•d^-1^) [2-AAF(L), *n* = 8] and CCl_4_ plus a high dose of 2-AAF (10 mg•kg^-1^•d^-1^) [2-AAF(H), *n* = 8]. An additional group of rats received equal quantities of subcutaneous olive oil and equal quantities of intragastric physiological saline as a control (normal group, *n* = 5). The 8wM group of rats were euthanized with pentobarbital sodium and euthanized at the end of the 8 weeks, and all others were euthanized at the end of the 12 weeks.

### Hepatic Hydroxyproline Content

Liver tissue (100 mg) was used for hydroxyproline (Hyp) determination, according to a modified method by Jamall et al. [12]. Briefly, liver tissue was homogenized and hydrolyzed in 6 N HCl at 110°C for 18 h. Chloramine T was then added after filtering the hydrolysate through a 0.45 mm Millipore filter (Millipore, Bedford, MA, USA). The mixture was then treated with 410 mM paradigm ethyl-amino-benzaldehyde and incubated at 60°C for 30 min. Samples were read at 560 nm until cooling to room temperature. The Hyp content of the liver, as an indirect measure of tissue collagen content, was expressed in milligram per gram of wet weight (mg/g).

### Histopathological and immunohistochemical analysis

Liver injury and cirrhosis were assessed using 4-μm-thick paraffin-embedded liver sections stained with hematoxylin and eosin (H&E) and Sirius red. Immunohistochemistry was carried out on paraffin-embedded or 7 μm-thick frozen slides, as previously described [13]. Briefly, sections were deparaffinized, washed, and preincubated in blocking solution, followed by incubation with primary antibodies. The primary antibodies were mouse monoclonal antibody against α-SMA (1:1000, Sigma-Aldrich, MO, USA); mouse monoclonal antibody against desmin (1:200, Epitomics Inc, CA, USA); rabbit polyclonal antibody against Col I (1:200, ProSci, CA, USA); rabbit polyclonal antibody against Col IV (1:200, Abcam, Cambridge, U.K.); mouse monoclonal antibody against CK19 (1:100, Proteintech Group Inc, IL, USA); mouse monoclonal antibody against OV6 (1:40, R&D Systems Inc, MN, USA); and mouse monoclonal antibody against CD68 (1:200, Hycult biotech, Uden, Netherlands). Sections were then incubated with HRP-conjugated secondary antibodies, washed, covered with DAB, and counterstained with hematoxylin. A Leica SCN 400 was used to scan the stained slides. 5 different views of 40 × 10 field per image were calculated the positive labelling area of every rat by the Image Pro Plus software for measuring OV6 positive labelling area.

### Double immunofluorescent analysis

To assess the differentiation of HPCs, double immunostainings, which were carried out on 7 μm-thick frozen slides, were performed to detect the co-expression of α-SMA (1:200) and OV6 (1:40). Alexa fluor 488 (1:2000) and cyanine 3 (1:2000) secondary antibodies (Jackson ImmunoResearch, West Grove, PA, USA) were used with counterstaining. Nuclei were stained with 49,6-diamidino-2-phenylindole (1:1000). Images were acquired using a laser scanning confocal microscope.

### Western Blot Analysis

The liver tissue was lysed by RIPA buffer with 1 mM PMSF and then homogenized in ice-cold water. 30 to 50 μg of total protein was used for immunoblot analysis, as previously described [14]. The following dilutions of primary antibodies were used: mouse monoclonal antibody to GAPDH (1:10,000, Chemicon International, CA, USA); α-SMA (1:1000); CD68 (1:1000); mouse monoclonal antibody to TNF-α (0.2 μg/ml, Millipore Corporation, MA, USA). The following second antibodies were used: IRDye 800CW Donkey anti-Mouse IgG (H + L) (1:10,000, LI-COR Bioscience, NE, USA), and IRDye 680RD Donkey anti-Rabbit IgG (H + L) (1:1000, LI-COR Bioscience). Band intensity was determined by scanning video densitometry.

### Real-Time Polymerase Chain Reaction Analysis

Total RNA was isolated from frozen hepatic tissue using Isogen (TOYOBO, Kita-ku, Osaka, Japan) [15]. qRT-PCR was performed using TaqMan One-Step RT-PCR Master Mix Reagents (TOYOBO) or the One-Step SYBR RT-PCR Kit (TOYOBO). GAPDH was employed as the housekeeping gene for qRT-PCR analysis. Primers used are listed in S1 Table.

### Cell Culture and TGF-β1 treatment of WB-F344 cells

The WB-F344 cells, a rat HPC cell line, were cultured in Dulbecco’s modified Eagle’s medium (DMEM) (Invitrogen, Carlsbad, CA) supplemented with 10% fetal bovine serum (FBS) (Invitrogen) at 37°C with 5% CO_2_. Cells were seeded on six-well Permanox Lab-Tek culture plates (1×10^5^ cells/well) and incubated for 24 hours. The media was then replaced by serum-free medium containing TGF-β1, at a final concentration of 2.5 ng/ml, and the cells were incubated for 24 hours and 48 hours after treatment. Control cells were treated with the same medium, but without TGF-β1.

### Small Molecule Inhibitor

Recombinant human WIF-1 (Wnt Inhibitor Factor-1, R & D Systems Inc., Minneapolis, MN, USA) were used at a concentration of 1ug/ml. Twenty-four hours after seeding the WB-F344 cells, the medium was replaced by serum-free medium, containing TGF-β1, with or without WIF-1, and the cells were incubated for 48 hours. Control cells were treated with the same medium, without TGF-β1 and WIF-1.

### Statistical Analysis

All data are presented as the mean ± SD. Statistical analyses were performed by analysis of variance (ANOVA) for multiple comparisons with SPSS10.0 software. *p* < 0.05 was considered to be statistically significant.

## Results

### 2-AAF promoted the inflammatory reaction in progressive cirrhosis induced by CCl_4_

**Fig 1:**
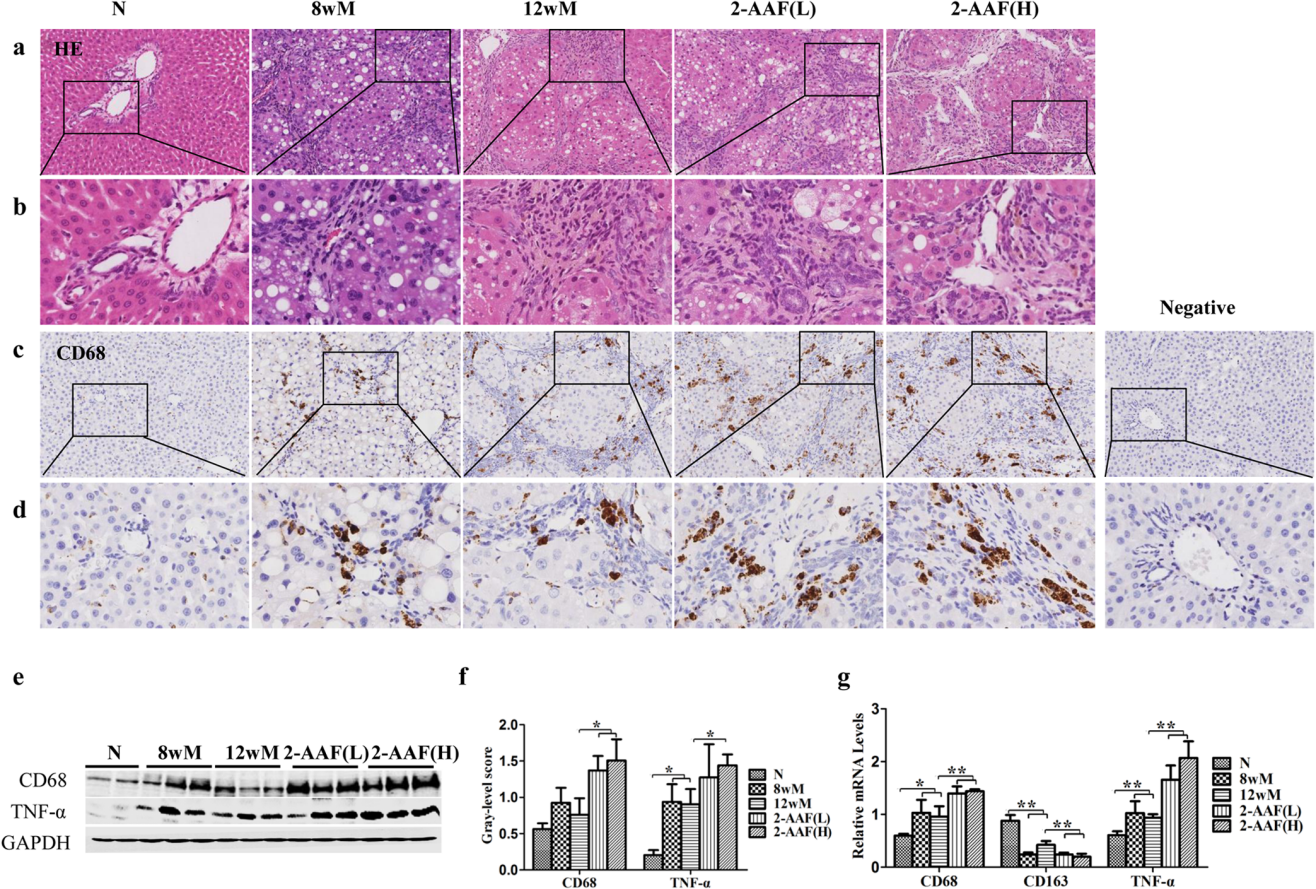
2-AAF promoted the inflammatory reaction. (a) H&E staining (×200). (b) A higher magnification of the squared area (×800). (c) CD68 immunostaining (×200) during cirrhosis progression. (d) A higher magnification of the squared area (×800). (e) Immunoblotting for CD68 and TNF-α. (f) The gray-level score indicates the immunoblotting histogram for CD68 and TNF-α. (g) Relative expression levels of CD68, CD163 and TNF-α were measured by qRT-PCR. *p < 0.05, **p < 0.01. Negative, Negative control; N, normal group; 8wM, the 8th week CCl4 group; 12wM, the 12th week CCl4 group.; 2-AAF(L), the 2-AAF(L) group; 2-AAF(H), the 2-AAF(H) group.

H&E staining showed that, after 12 weeks of CCl_4_ treatment, the hepatic lobular structure was disorganized with moderate hepatocellular steatosis and ballooning in close proximity to inflammatory cells. Hepatic centrilobular injury was similar in the 2-AAF(L) and 2-AAF(H) groups to that observed in the 12wM group. However, an expansion of basophilic cells, was observed in both the 2-AAF(L) and 2-AAF(H) groups and was more prominent in the 2-AAF(H) group. These cells had a high nuclear to cytoplasmic ratio and were detected in the periportal areas, with some extending deeply into the lobule and formed porto-portal and porto-central bridges (Fig 1A and 1B).

The immunostaining showed that CD68-positive cells were present in hepatic sinusoids in the normal group and were mainly located in the portal areas and fibrotic septa in the 12wM group. In the 2-AAF(L) and 2-AAF(H) groups, CD68-positive cells appeared not only in portal areas and adjacent to fibrotic septa, but also infiltrated the lobule (Fig 1C and 1D). In the 8wM and 12wM groups, the protein expressions of CD68 and TNF-α were higher compared to the normal group (TNF-α *p* <0.05) and even significantly higher in the 2-AAF(L) and 2-AAF(H) groups compared with the 12wM group (CD68 *p* <0.05; TNF-α, 2-AAF(H) *p* <0.05) (Fig 1E and 1F). The increased expressions of CD68 and TNF-α were confirmed by qRT-PCR analysis (Fig 1G). However, CD163 mRNA expression was decreased after administration of 2-AAF (*p* <0.01) (Fig 1G). These results suggest that 2-AAF promotes the inflammatory reaction of the liver through the activation of pro-inflammatory macrophages.

### 2-AAF advanced cirrhosis progression induced by CCl_4_

**Fig 2:**
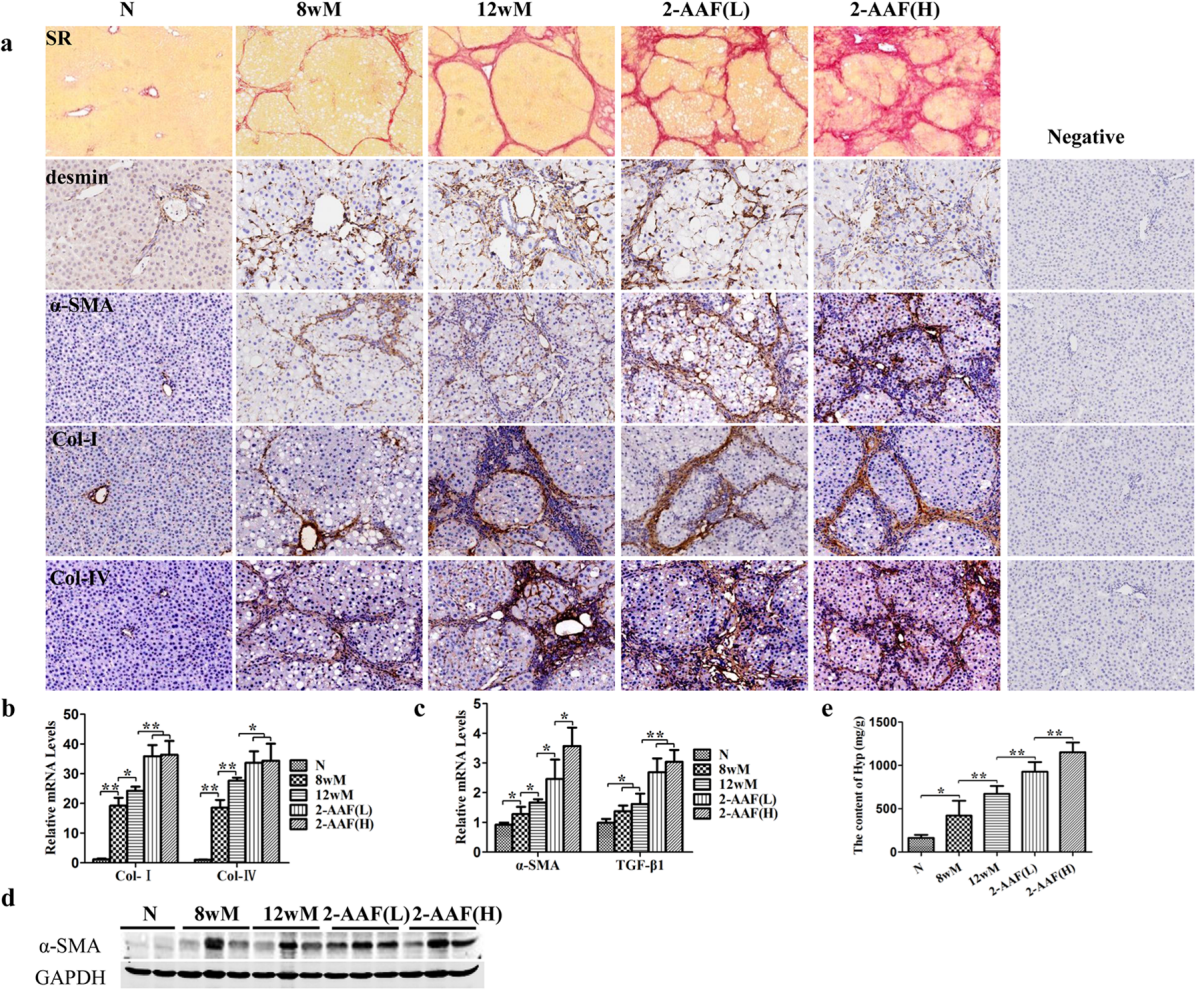
2-AAF advanced the progress of cirrhosis. (a) Sirius Red Staining (100×) and immunohistochemistry of desmin, α-SMA, Col I and Col IV (×200). (b) The relative expression levels of Col I and Col IV were measured by qRT-PCR. (c) The relative expression levels of α-SMA and TGF-β1 were measured by qRT-PCR. (d) Western blot of α-SMA. (e) The hydroxyproline content of liver tissue. *p < 0.05, **p < 0.01.

Sirius red staining showed liver injury, indicating early stage cirrhosis, in the 8wM group. In the 12wM group, there was complete interconnection between central veins, dividing the parenchyma into pseudo-lobules centered by a portal tract. In the 2-AAF(L) and 2-AAF(H) groups, the fibrotic septa was thickened, had progressed into the lobule and many nodules were surrounded by broad septa. In some areas, extensive tracts of fibrous tissue, containing a few larger nodules and small regions of fragmented nodules, were observed. This was especially evident in the 2-AAF (H) group (Fig 2A).

Immunostaining showed α-SMA, desmin, Col I and Col IV expression located mainly in the fibrotic septa (Fig 2A). Interestingly, in the 2-AAF(L) and 2-AAF(H) groups, α-SMA-positive cells infiltrated the lobule. Moreover, α-SMA protein and mRNA expression was significantly increased in the 2-AAF(L) and 2-AAF(H) groups, compared to the 12wM group (*p* < 0.05) and was higher in the 2-AAF(H) group than in the 2-AAF(L) group (*p* < 0.05) (Fig 2C and 2D). In addition, Col I and Col IV mRNA levels were significantly higher in the 2-AAF(L) and 2-AAF(H) groups compared with the 12wM group (Col I *p* < 0.01, Col IV *p* < 0.05) (Fig 2B). TGF-β1 is considered to be the most powerful fibrogenic cytokine and is mainly produced by myofibroblasts [16]. Results of the current study showed that the expression of TGF-β1 increased in the 8wM and 12wM groups compared to the normal group (*p* < 0.05). In the 2-AAF(L) and 2-AAF(H) groups, TGF-β1 expression was significantly higher than that in the 12wM group (*p* < 0.01) (Fig 2C). The Hyp content of liver tissue significantly higher in the 8wM and 12wM groups compared with the normal group (8wM *p*<0.05, 12wM *p*<0.01). After administration of 2-AAF, Hyp content was significantly higher than in the 12wM group (*p*<0.01). Furthermore, Hyp content was higher in the 2-AAF(H) group compared to the 2-AAF(L) group (*p*<0.01) (Fig 2E). Thus, the expression profile clearly indicate that progressive cirrhosis was enhanced after administration of 2-AAF.

### 2-AAF promoted the differentiation of HPCs to myofibroblasts

**Fig 3:**
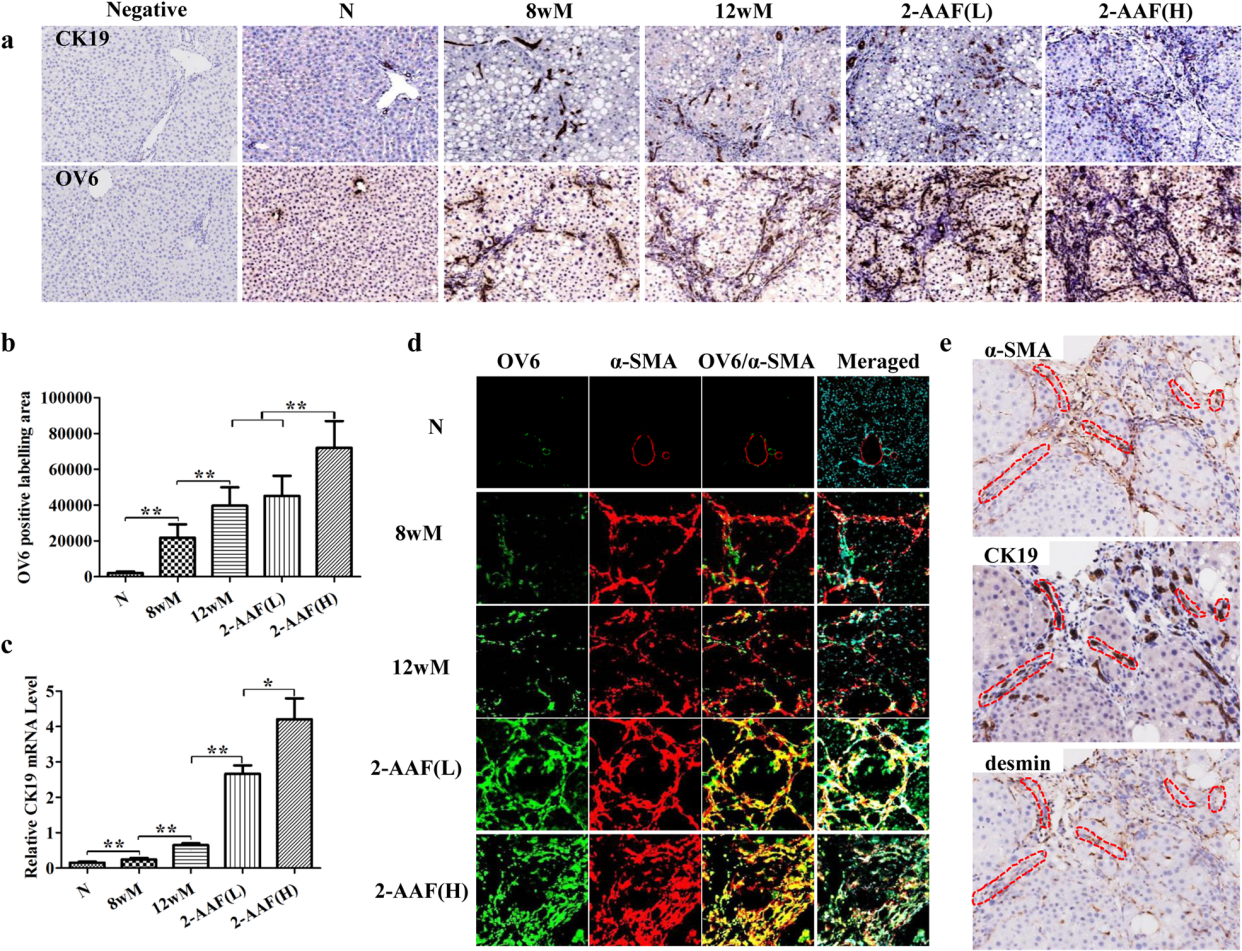
2-AAF promoted the activation and expansion of HPCs, and promoted the differentiation of HPCs to myofibroblasts. (a) Immunohistochemistry of CK19 and OV6 (×200). (b) The change in the OV6-positive labeling area. (c) The relative expression level of CK19 was measured by qRT-PCR. (d) Double immune-staining of α-SMA (red), OV6 (green) and DAPI (blue), merged α-SMA, OV6 and DAPI (×200). (e) Serial section immunostaining of α-SMA, CK19 and desmin in the 2-AAF(H) group (×200). *p < 0.05, **p < 0.01.

Immunostaining showed that the commonly used rodent HPC markers, CK19 and OV6 [17, 18], were stained in the bile ducts of normal rats. In the CCl_4_-treated rats, CK19- and OV6-positive cells formed duct-like structures surrounding the portal tract and in fibrotic septa. As expected, in the 2-AAF(L) and 2-AAF(H) groups, CK19- and OV6-positive cells extended across the liver lobule, forming bridges between portal tracts. Expansion of CK19- and OV6-positive cells connected portal and centrilobular areas, dividing the parenchyma into smaller pseudo-lobules, especially in the 2-AAF(H) group. CK19- and OV6-positive cells formed clusters that contained a high number of cells inside the lobule (Fig 3A). The number of OV6-positive cells increased during the period of CCl_4_ treatment (*p* < 0.01). In the 2-AAF(H) group, the area of OV6-positive cells was significantly higher compared to the 12wM group and the 2-AAF (L) group (*p* < 0.01) (Fig 3B). In addition, the CK19 mRNA expression was significantly higher in the 2-AAF(L) and 2-AAF(H) groups compared to the 12wM group (*p* < 0.01). The CK19 mRNA level was also higher in the 2-AAF(H) group than in the 2-AAF(L) group (*p* < 0.05) (Fig 3C). These results demonstrate that a large number of HPCs were activated after administration of 2-AAF and indicate a correlation between the magnitude of HPC expansion and the dose of 2-AAF administered.

To better elucidate the differentiation and activation of HPCs, co-localization among OV6 and α-SMA was detected by confocal microscopy. The results indicated that few cells, which were positive for both OV6 and α-SMA, were observed in the normal liver and a number of cells, which were positive for both OV6 and α-SMA were detected in the 8wM and 12wM groups. OV6 was detected in the fibrotic septa, extended into the liver lobule and was largely co-localized with α-SMA-positive cells in the 2-AAF(L) and 2-AAF(H) groups, and the number of double positive cells had obviously increased compared to the 12wM group (Fig 3D, S2 Fig). Serial section immunostaining did not show any expression of desmin in CK19-positive HPCs, even in the 2-AAF(H) group, but did show co-expression of α-SMA in CK19-positive HPCs (Fig 3E). Taken together, these observations indicate that administration of 2-AAF in progressive cirrhosis caused activation of HPCs, which, mainly differentiated into α-SMA-positive myofibroblasts, thus promoting the fibrogenic process.

### The non-canonical Wnt signaling pathway was involved in HPC expansion and differentiation

**Fig 4:**
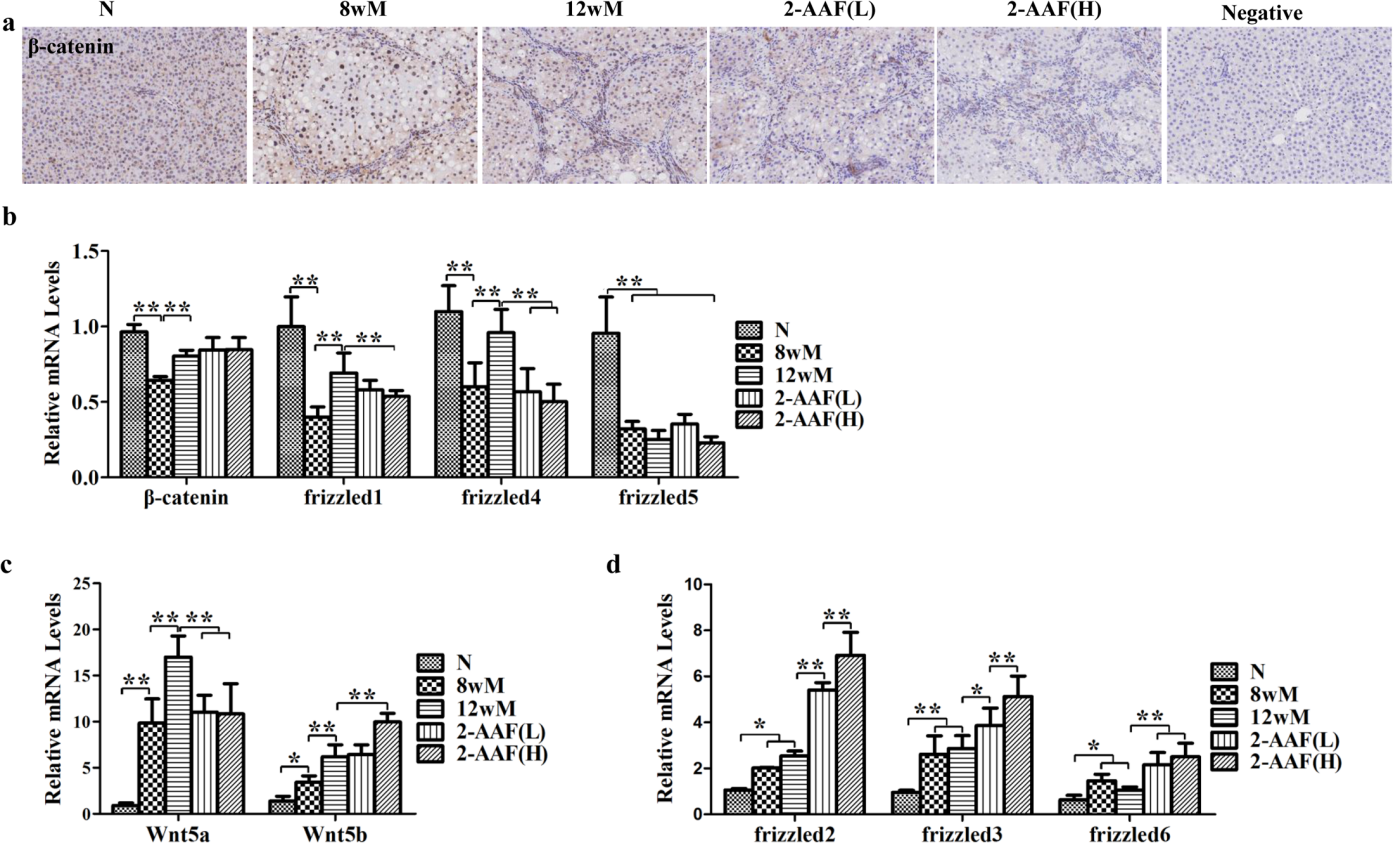
The non-canonical Wnt signaling pathway is involved in HPCs expansion and differentiation. (a) β-catenin immunostaining (×200). (b) Expressions of β-catenin, frizzled1, frizzled4 and frizzled5, representing the canonical Wnt signaling, were measured by qRT-PCR. (c) Expression levels of Wnt5b and Wnt5a, representing the non-canonical Wnt pathway, were measured by qRT-PCR. (d) Expression levels of receptors, frizzled2, frizzled3 and frizzled6, were measured by qPCR. *p < 0.05, **p < 0.01.

From the immunohistochemistry results, the location of β-catenin protein was not obviously different among the groups (Fig 4A). As shown in Fig 4B, there was no obvious difference in the mRNA level of β-catenin between the 12wM group and the 2-AAF(L) and 2-AAF(H) groups. The expressions of the canonical Wnt pathway receptors, frizzled1 and frizzled4, were remarkably decreased after administration of 2-AAF (*p* < 0.01), though the frizzled5 mRNA level was not significantly changed, suggesting that the canonical Wnt signaling pathway was inactivated in the 2-AAF groups.

The non-canonical Wnt signaling pathway ligands, Wnt5b and Wnt5a, were increased in the 8wM and 12wM groups compared with the normal group (Wnt5a, *p* < 0.01; Wnt5b, *p* < 0.05, *p* < 0.01). After administration of 2-AAF, Wnt5a expression was significantly lower (*p* < 0.01); however, Wnt5b expression was significantly higher in the 2-AAF(H) group than in the 12wM group (*p* < 0.01) (Fig 4C). In the 8wM and 12wM groups, expression of the non-canonical Wnt pathway receptors, frizzled2, frizzled3 and frizzled6, was higher compared to the normal group (frizzled2 *p*<0.05, frizzled3 *p*<0.01, frizzled6 *p*<0.05). Furthermore, expression of these receptors was higher in the 2-AAF(L) and 2-AAF(H) groups than in the 12wM group (frizzled2 *p* < 0.01; frizzled3 2-AAF(L) *p* < 0.05 and 2-AAF(H) *p* < 0.01; frizzled6 *p* < 0.01). Moreover, in the 2-AAF(H) group, frizzled2 and frizzled3 expression was higher than in the 2-AAF(L) group (*p* < 0.01) (Fig 4D). These results demonstrate that the non-canonical Wnt signaling pathway was activated during the differentiation of HPCs into myfibroblasts.

### Non-canonical Wnt signaling pathway was activated during the specification of WB-F344 into myfibroblasts

**Fig 5:**
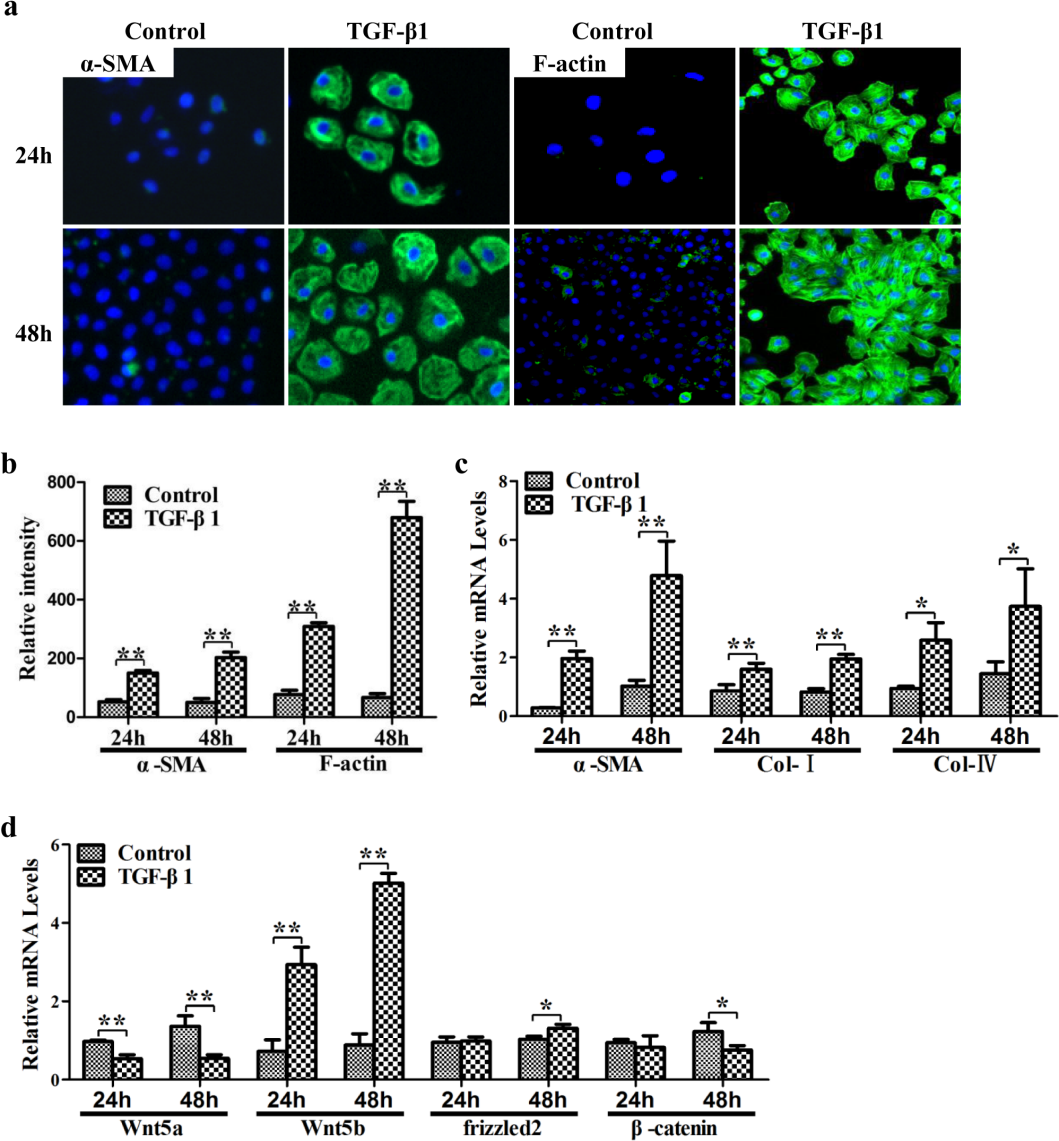
Non-canonical Wnt signaling pathway was activated during the specification of WB-F344 cells into myfibroblasts. (a) Immunostaining of α-SMA and F-actin. (b) The fluorescent intensity of α-SMA and F-actin. (c) Expression levels of α-SMA, Col I and Col IV. (d) Wnt5a, Wnt5b, frizzled2 and β-catenin were determined by qRT-PCR. *p < 0.05, **p < 0.01.

To further investigate whether the non-canonical Wnt signaling pathway is activated during the specification of HPCs into myfibroblasts, WB-F344 cells were treated with or without TGF-β1 for 24 hours and 48 hours. Immunostaining showed that, after treatment with TGF-β1, the cells expressed substantial amounts of α-SMA and F-actin (Fig 5A), and the fluorescent intensity of α-SMA and F-actin was clearly increased in the TGF-β1-treated cells compared to control cells (*p* < 0.01) (Fig 5B). In addition, the mRNA levels of α-SMA, Col I and Col IV were significantly increased after exposure to TGF-β1 for 24 hours (*p* < 0.01, *p* < 0.01 and *p* < 0.05) and 48 hours (*p* < 0.01, *p* < 0.01 and *p* < 0.05) (Fig 5C).

The mRNA expression of β-catenin was slightly decreased after 24 hours of TGF-β1 treatment and was significantly decreased after for 48 hours of TGF-β1 treatment (*p* < 0.05) (Fig 5D). Consistent with the *in vivo* results, Wnt5b mRNA expression was significantly increased in TGF-β1-treated cells compared to control cells (24 hours *p* < 0.01, 48 hours *p* < 0.01) and the frizzled2 mRNA level was significantly increased after 48 hours of TGF-β1 treatment (*p* < 0.05). However, Wnt5a mRNA expression was significantly decreased compared to cells not treated with TGF-β1 (24 hours *p* < 0.01, 48 hours *p* < 0.01). These results indicate activation of non-canonical signaling pathway and inhibition of canonical signaling in WB-F344 stimulated with TGF-β1.

### Blocking the non-canonical Wnt signaling pathway can inhibit the specification of WB-F344 into myfibroblasts

**Fig 6:**
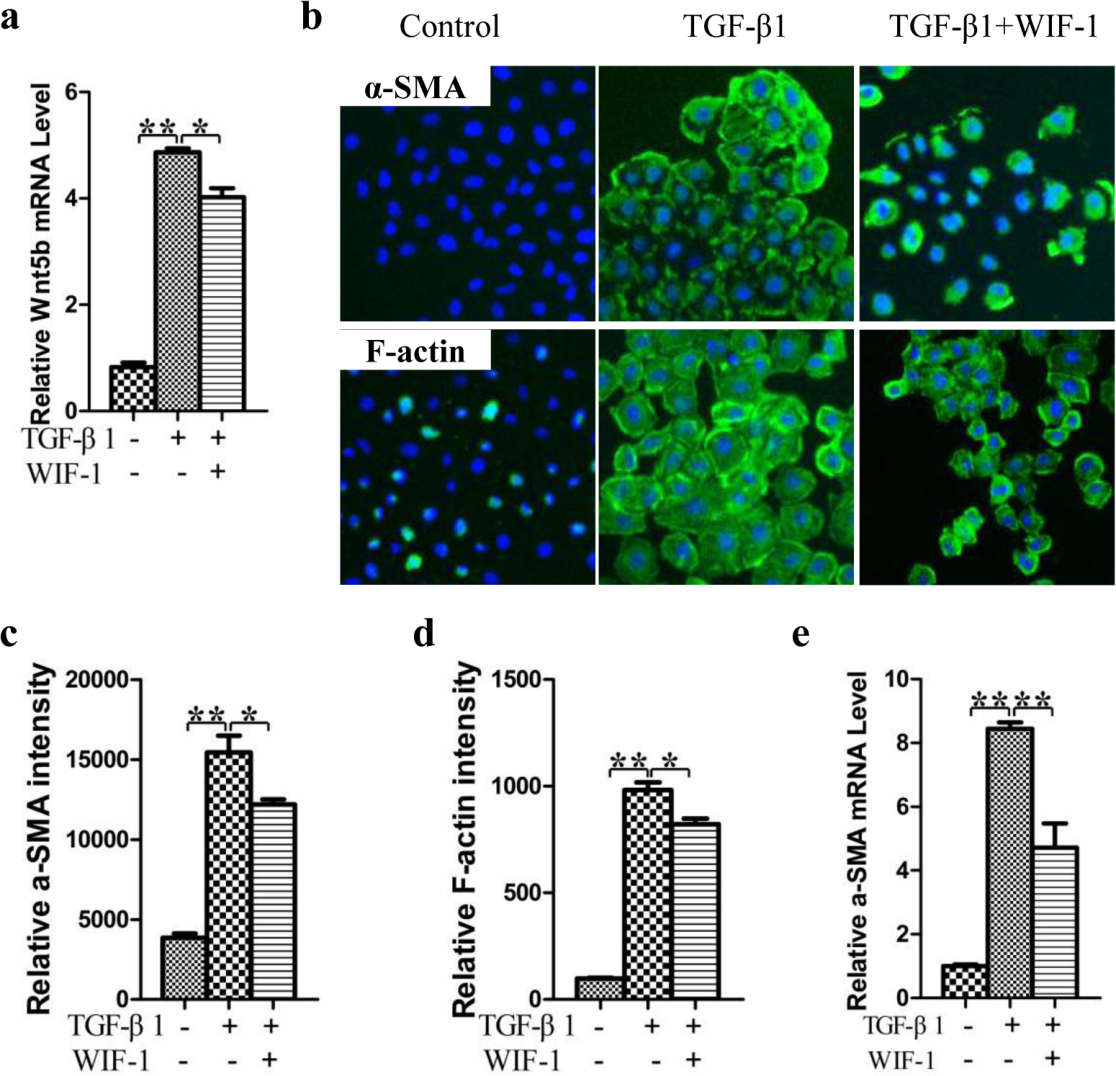
Blocking the non-canonical Wnt pathway can inhibit the specification of WB-F344 cells into myfibroblasts. (a) mRNA expression of Wnt 5b. (b) Immunostaining of α-SMA and F-actin. (c) The fluorescent intensity of α-SMA. (d) The fluorescent intensity of F-actin. (e) α-SMA was determined by qRT-PCR. *p < 0.05, **p < 0.01.

In order to further investigate whether the non-canonical Wnt signaling pathway regulates the specification of WB-F344 cells into myfibroblasts, cells were treated with TGF-β1 with or without WIF-1 for 48 hours. The mRNA expression of Wnt5b was significantly decreased after treatment with WIF-1 for 48 hours (*p* < 0.05) (Fig 6A), indicating that the non-canonical Wnt pathway was inhibited after WIF-1 treatment. Immunostaining showed that, the a-SMA and F-actin expression in WIF-1-treated cells was down regulated (Fig 6B) and the fluorescent intensity of α-SMA and F-actin was clearly decreased in the WIF-1-treated cells compared to no-WIF-1- treated cells (*p* < 0.05) (Fig 6C). Consistent with immunostaining results, qPCR showed that the a-SMA expression was significantly decreased in the WIF-1-treated cells (*p* < 0.01) (Fig 6D). These results demonstrate that inhibition of the non-canonical Wnt pathway can prevent the specification of WB-F344 cells into myfibroblasts.

## Discussion

When proliferation of hepatocytes is impaired, the loss of the ability of “mature” hepatocytes to proliferate and restore the liver after injury or partial removal, appears to be compensated for by involvement of HPCs, which proliferate and differentiate into ductular cells or hepatocytes [19]. 2-AAF combined with CCl_4_ is a classic experimental model to study HPC-dependent regeneration in rats [20, 21]. In this study, subcutaneous injection of CCl_4_ (50%) was used to induce cirrhosis for 8 weeks, after which, a low-dose of CCl_4_ (30%) was administered for 4 weeks to maintain the progression of cirrhosis. The effect on HPCs was investigated in this cirrhosis model by administering 2-AAF to inhibit hepatocyte regeneration and to increase the HPC expansion. The results showed that the expression of HPC markers, OV6 and CK19, significantly increased during cirrhosis progression. In the CCl_4_/2-AAF treated rats, OV6- and CK19-positive cells extended across the liver lobule, formed bridges between portal tracts and divided the parenchyma into smaller pseudo-lobules. Our results demonstrated HPC activation after administration of 2-AAF and a correlation between the magnitude of HPC expansion and the dose of 2-AAF administered.

In both human and murine studies, HPC proliferation has been suggested to be accompanied by matrix remodeling, which can result in fibrosis if unbalanced and uncontrolled [16, 22]. Previous studies of chronically diseased livers have demonstrated a correlation between HPC proliferation and the severity of cirrhosis [23, 24]. Results of the current study revealed that the 2-AAF/CCl4 –induced cirrhosis model led to continuous injury, parechymal cell damage and advanced progression of cirrhosis after administration of 2-AAF. The severity of cirrhosis was associated with the increase in the number of HPCs. The combination of CCl_4_ and 2-AAF led to extensive cirrhosis with marked HPC expansion, whereas CCl_4_ alone caused moderate cirrhosis. Low doses of 2-AAF generated low toxicity, as indicated by only a slight increase in ECM induction, while high 2-AAF doses generated excess ECM deposition as revealed by Sirius red staining and Hyp content. These results were confirmed by immunostaining and qRT-PCR.

Macrophages play a central role in the inflammation response and fibrogenic processes in the liver. There appear to be two distinct types of macrophages: M1 (CD68-positive, pro-inflammatory macrophage) [25] and M2 (CD163-positive, anti-inflammatory macrophage) [26]. It has been suggested that TNF-α is mainly secreted by M1 macrophages after liver injury [27]. This study showed that expressions of CD68 and TNF-α were increased; however, the expression of CD163 was decreased after administration of 2-AAF. These results suggest that pro-inflammatory macrophages were activated and released large amounts of TNF-α after administration of 2-AAF, promoting an inflammatory reaction during the process of cirrhosis. It has been suggested that several macrophage-produced cytokines play roles in mediating the growth of HPCs [28] and TNF-α appears to play a critical role in mediating HPCs proliferation and differentiation [29, 30]. These published data and results of the current study support the hypothesis that interactions with hepatic macrophages and HPCs are probably necessary for proliferation and the effects on differentiated HPCs in the pathological microenvironment of progressive cirrhosis, however further research is needed to confirm this.

HPCs are able to differentiate to hepatocytes or ductular cells when the proliferation of mature hepatocytes is inhibited or blocked [31]. However, some researchers have found co-expression of epithelial and mesenchymal markers in HPCs and have demonstrated that HPCs have an ability to transdifferentiate to hepatic stellate cells or myofibroblasts [32–34]. Whether the cells co-expressing epithelial and mesenchymal markers are activated HSCs or myofibroblasts, or whether these cells arise from mesenchymal progenitor or an epithelial-mesenchymal transition between HPCs and HSCs, remains to be determined. In this study, double immunostaining results showed that OV6-positive cells were largely co-localized with α-SMA-positive cells after administration of 2-AAF, demonstrating that proliferating HPCs can mainly transdifferentiate into myofibroblasts and exhibit a profibrotic effect in the pathological microenvironment of progressive cirrhosis. Interestingly, in the 2-AAF(H) group, serial section immunostaining did not show any desmin expression in CK19-positive HPCs, but did show α-SMA expression in CK19-positive HPCs. These results indicate that HPCs do not transdifferentiate to hepatic stellate cells, but have an ability to transdifferentiate into α-SMA-positive myofibroblasts in progressive cirrhosis.

Additionally, the expression of TGF-β1 coincided with HPC expansion during the process of cirrhosis after administration of 2-AAF. TGF-β1 is a well-known EMT inducer [35] and a major fibrogenic cytokine, which is produced in abundance during liver fibrosis and cirrhosis [16, 36]. Moreover, the HPC phenotype changed from epithelial cells to mesenchymal-like cells after TGF-β1 treatment [37]. Therefore, the fate change of the HPC line (WB-F344) was investigated after treated with TGF-β1. WB-F344 responded to stimulation with TGF-β1 and the expressions of α-SMA and F-actin, as well as the ECM genes, Col I and Col IV were enhanced. These results also suggest that HPCs have the ability to transdifferentiate into myofibroblasts in the appropriate environment.

Wnt pathways have been classified as either canonical or non-canonical signaling pathways [38]. Wnt1, Wnt3a and Wnt8 are more commonly encountered in canonical signaling, while Wnt5a, Wnt5b and Wnt11 are predominantly involved in non-canonical signaling [39]. The study of the stem cell microenvironment has indicated that Wnt signaling pathways are important for the regulation of stem cell proliferation and differentiation towards committed lineages [40]. The canonical Wnt pathway has been extensively studied in HPC expansion and differentiation towards hepatocytes [41–43]. However, the function of non-canonical Wnt pathways in HPC expansion and differentiation remains unknown. In this study, it was found that the expressions of Wnt5b, frizzled2, frizzled3 and frizzled6 were markedly increased after administration of 2-AAF, but the mRNA expression of β-catenin was not changed by administration of 2-AAF, suggesting that the activation of Wnt pathway is not mediated through the β-catenin pathway. Further investigation of the non-canonical Wnt pathway was performed by investigating the transdifferentiation of WB-F344 cells into myofibroblasts, *in vitro*. As expected, both Wnt5b and frizzled2 expression were significantly up regulated in WB-F344 cells after treatment with TGF-β1 and the β-catenin level was significantly decreased after 48 hours of treatment with TGF-β1. Blocking the non-canonical Wnt pathway with WIF-1 led to significant down regulation of Wnt5b expression and significant decreases in both a-SMA and F-actin expression in WB-F344 cells. Thus, these *in vitro* results confirmed the *in vivo* findings and demonstrated that the non-canonical Wnt pathway was activated during the process of HPC differentiation into myofibroblasts, whereas, canonical Wnt signaling was inhibited, and blocking the non-canonical Wnt pathway inhibited the differentiation of HPCs into myofibroblasts.

In conclusion, results of this study demonstrated HPC activation and proliferation in progressive cirrhosis induced by 2-AAF/CCl_4_. Furthermore, HPCs mainly transdifferentiated into α-SMA-positive myofibroblasts, while exhibiting a profibrotic effect through activation of the non-canonical Wnt pathway in progressive cirrhosis induced by 2-AAF/CCl_4_. Blocking the non-canonical Wnt signaling pathway in WB-F344 cells can inhibit differentiation into myofibroblasts. Thus, blocking the non-canonical Wnt signaling pathway may change the fate of HPCs, consequently suppressing the progression of cirrhosis. Therefore, this may represent an initial step toward developing a potential therapeutic target for the treatment of liver diseases in humans.

## Supporting Information

S1 Fig Double immune-staining of CK19 (red), OV6 (green) and DAPI (blue), merged CK19, OV6 and DAPI (×200). (TIF)

S2 Fig The higher magnification of double immune-staining of α-SMA (red), OV6 (green), and DAPI (blue), merged α-SMA, OV6, and DAPI. (TIF)

S1 Table Primer Pairs and Probes Used for Real-time PCR. Abbreviations: GAPDH, glyceraldehyde-3-phosphate dehydrogenase; a-SMA, alpha smooth muscle actin; CK19, cytokeratin 19.
(DOC)
