# Supplementary figures and images for: Hepatic Progenitor Cells Contribute to the Progression of 2-Acetylaminofluorene/Carbon Tetrachloride-Induced Cirrhosis via the Non-Canonical Wnt Pathway
Source: PLoS One. 2015 Jun 18;10(6):e0130310. doi: 10.1371/journal.pone.0130310 (PMC4473299; doi:10.1371/journal.pone.0130310)

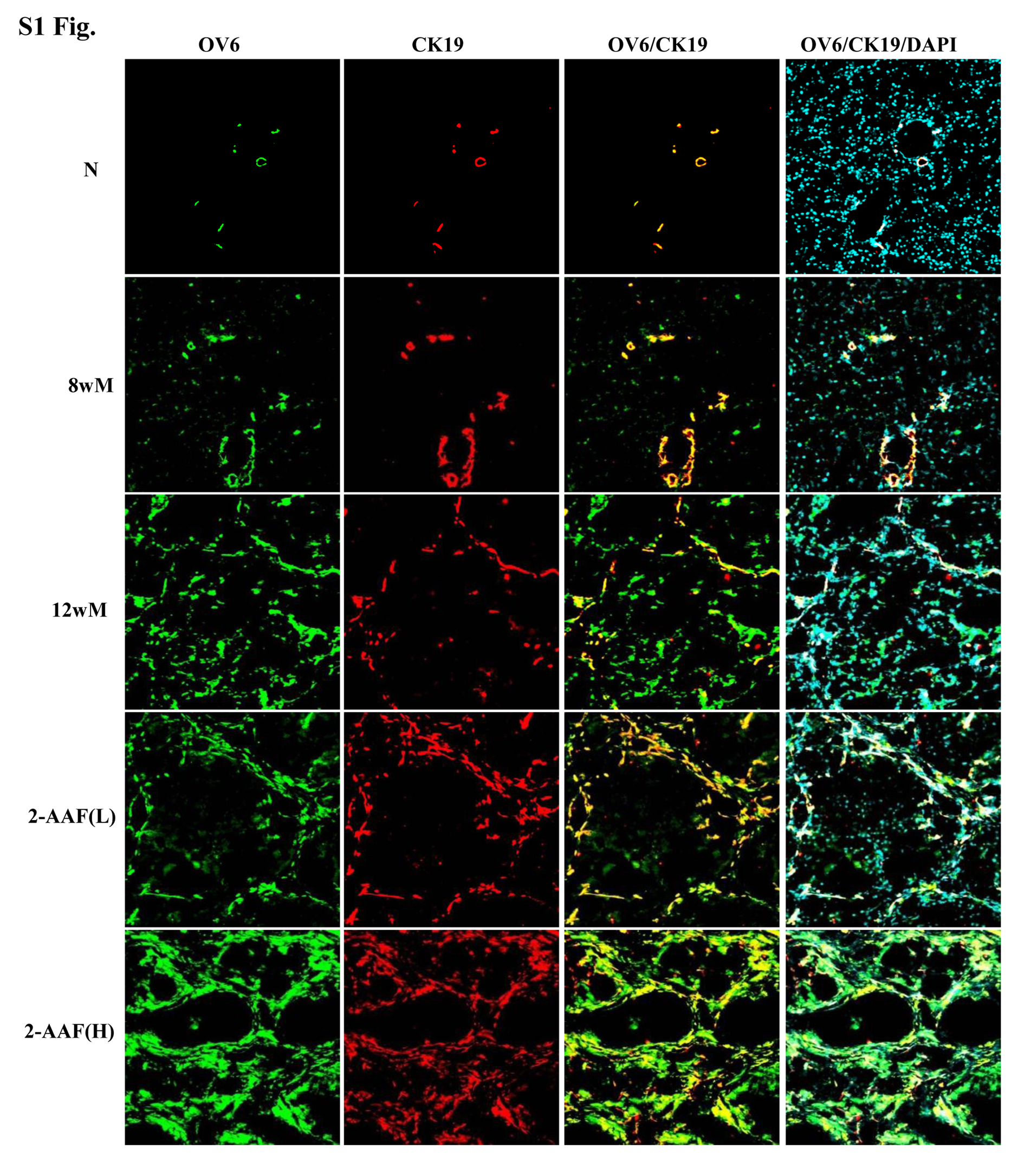

Supplement: S1 Fig — (TIF) [file pone.0130310.s001.tif]

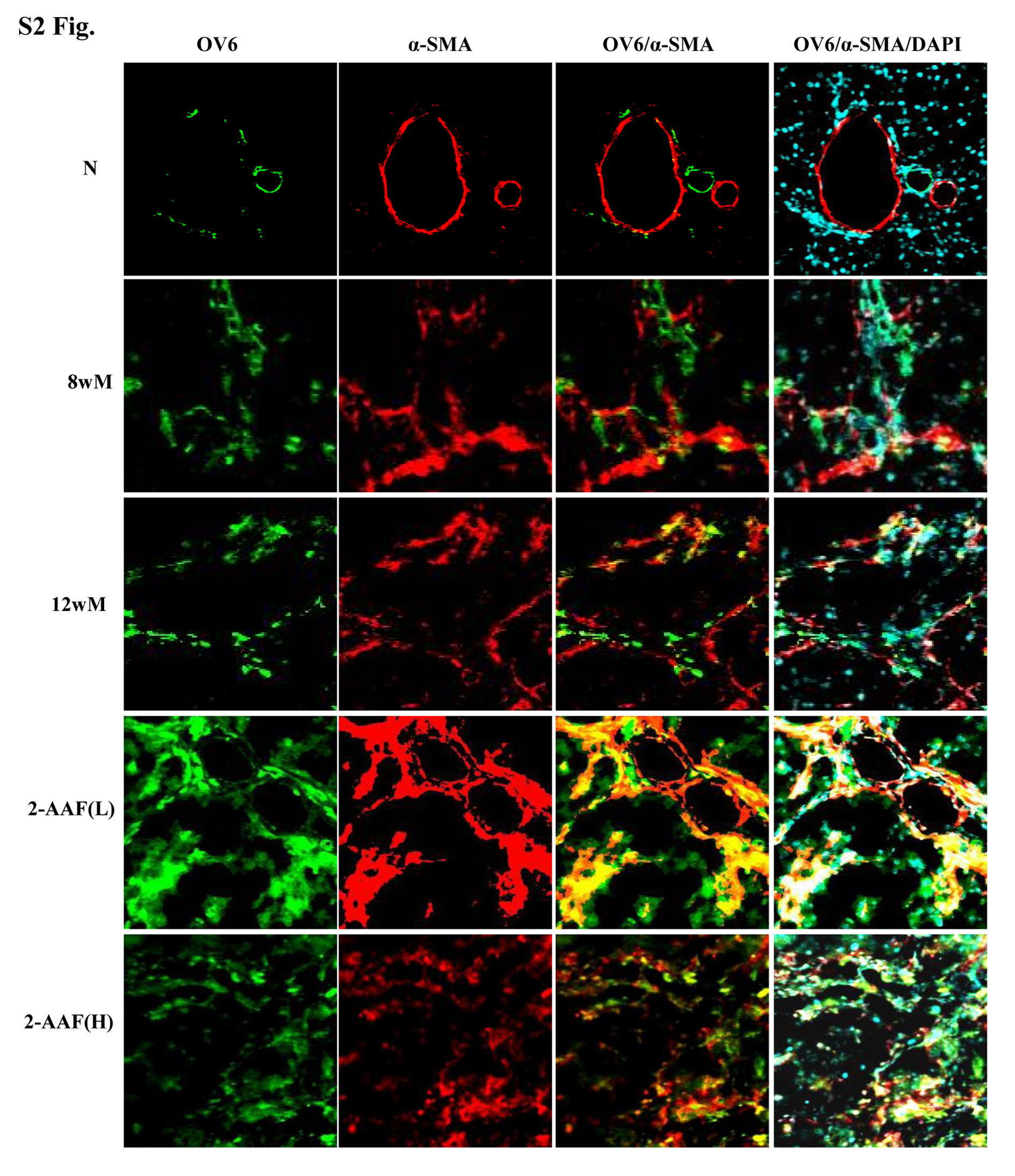

Supplement: S2 Fig — (TIF) [file pone.0130310.s002.tif]
