# Supplementary material for: Hepatic Progenitor Cells Contribute to the Progression of 2-Acetylaminofluorene/Carbon Tetrachloride-Induced Cirrhosis via the Non-Canonical Wnt Pathway
Source: PLoS One. 2015 Jun 18;10(6):e0130310. doi: 10.1371/journal.pone.0130310 (PMC4473299; doi:10.1371/journal.pone.0130310)
Supplement: S1 Table — Abbreviations: GAPDH, glyceraldehyde-3-phosphate dehydrogenase; a-SMA, alpha smooth muscle actin; CK19, cytokeratin 19. (DOC) [file pone.0130310.s003.doc]

**S1 Table**. Primer Pairs and Probes Used for Real-time PCR

| *Primer name* | *Sequence* | | *Note* |
| --- | --- | --- | --- |
| *α-SMA* |  | | TaqMan |
| Forward | 5'-GAGGAGCATCCGACCTTGC-3' | |  |
| Reverse | 5'-TTTCTCCCGGTTGGCCTTA-3' | |  |
| Probe | 5'-AACGGAGGCGCCGCTGAACC-3' | |  |
| *collagen I* |  | | TaqMan |
| Forward | 5'-GCCTCCCAGAACATCACCTA-3' | |  |
| Reverse | 5'-CCTTCTTGAGGTTGCCAGTC-3' | |  |
| Probe | 5'-AAGAACAGCGTGGCCTACAT-3' | |  |
| *collagen IV* |  | | TaqMan |
| Forward | 5'-GAGAGAGGCTTCCCTGGTCT-3' | |  |
| Reverse | 5'-ACTTGCTCCAGAGGGACCTT-3' | |  |
| Probe | 5'-TCTGGTGAACCTGGCAAAC-3' | |  |
| *β-catenin* | |  | SYBR |
| Forward | | 5’-GTCTGAGGACAAGCCACAGGACTAC-3’ |  |
| Reverse | | 5’- AATGTCCAGTCCGAGATCAGCA -3’ |  |
| *Frizzled 1* | |  | SYBR |
| Forward | | 5’- GGGAATGCAGTCACCAGTACCA-3’ |  |
| Reverse | | 5’- CCAGACCCATAGCAGGTTCCA-3’ |  |
| *Frizzled 2* | |  | SYBR |
| Forward | | 5’- ACTGCAAGAGCCTAGCCATCC -3’ |  |
| Reverse | | 5’- ATCCAGAAGCCCGACGTGA-3’ |  |
| *Frizzled 3* | |  | SYBR |
| Forward | | 5’- ACACATGGCACCAGCATGAAC -3’ |  |
| Reverse | | 5’- CCATGCGAAGGCCAAGACTAA-3’ |  |
| *Frizzled 4* | |  | SYBR |
| Forward | | 5’- GACAACTTTCACGCCGCTCA-3’ |  |
| Reverse | | 5’- TTCAGGACTGGTTCACATCGTCTC-3’ |  |
| *Frizzled 5* | |  | SYBR |
| Forward | | 5’- CGAGAGCACAGCCACATTCACTA -3’ |  |
| Reverse | | 5’- GAGCTGGCCATGCCAAAGA-3’ |  |
| *Frizzled 6* | |  | SYBR |
| Forward | | 5’- CAGCAGCGTCCAACTCCAAG -3’ |  |
| Reverse | | 5’- TGCACTCCATCAGGCCAGTC-3’ |  |
| *Wnt 5a* | |  | SYBR |
| Forward | | 5'- GCGCTGCTGGAGTGGTAAAT-3' |  |
| Reverse | | 5'- AGCCAGTCCCGAGGTAAGTC-3' |  |
| *Wnt 5b* | |  | SYBR |
| Forward | | 5’- CGAGCCCTCATGAACTTACAGAAC -3’ |  |
| Reverse | | 5’- GGAGACTCCGTGACATTTGCAG -3’ |  |
| *GAPDH* | |  | SYBR |
| Forward | | 5'-GGCACAGTCAAGGCTGAGAAT G-3' |  |
| Reverse | | 5'-ATGGTGGTGAAGACGCCAGTA-3' |  |
| *GAPDH* | |  | TaqMan |
| Forward | | 5'-AAGATGGTGAAGGTCGGTGTG-3' |  |
| Reverse | | 5'-GAAGGCAGCCCTGGTAACC-3' |  |
| Probe | | 5'-CGGATTTGGCCGTATCGGACGC-3' |  |
| *TNF-α* | |  | TaqMan |
| Forward | | 5'-GCTCCCTCTCATCAGTTCCATG-3' |  |
| Reverse | | 5'-TACGGGCTTGTCACTCGAGTTTTG-3' |  |
| Probe | | 5'-CCCAGACCCTCACACTCAGATCATCTT C-3' |  |
| *TGF-β1* | |  | TaqMan |
| Forward | | 5'-TGCTTCCGCATCACCGT-3' |  |
| Reverse | | 5'-TAGTAGACGATGGGCAGTGGC-3' |  |
| Probe | | 5'-CTGCGTGCCGCAGGCTTTGG-3' |  |
| *CK19* | |  | *SYBR* |
| Forward | | 5'-TATCTGGATCTGCGTAGTGTGG-3' |  |
| Reverse | | 5'-ATACAAAACCAAACTGGGGAT G-3' |  |

Abbreviations: GAPDH, glyceraldehyde-3-phosphate dehydrogenase; a-SMA, alpha smooth muscle actin; CK19, cytokeratin 19.
